# Non-Coding RNAs in CD4^+^ T Cells: New Insights Into the Pathogenesis of Systemic Lupus Erythematosus

**DOI:** 10.3389/fimmu.2020.00568

**Published:** 2020-04-03

**Authors:** Xiaofei Gao, Limin Liu, Xiaoli Min, Sujie Jia, Ming Zhao

**Affiliations:** ^1^Hunan Key Laboratory of Medical Epigenomics, Department of Dermatology, The Second Xiangya Hospital of Central South University, Changsha, China; ^2^Department of Pharmacy, The Third Xiangya Hospital, Central South University, Changsha, China

**Keywords:** non-coding RNAs, CD4^+^ T cells, T helper cells, biomarkers, systemic lupus erythematosus

## Abstract

Non-coding RNAs (ncRNAs) are indispensable for CD4^+^ T cell differentiation and functions. By directly or indirectly regulating immune gene expression, ncRNAs give flexible instructions to guide the biological processes of CD4^+^ T cells and play a vital role in maintaining immune homeostasis. However, the dysfunction of ncRNAs alters the gene expression profiles, disturbs the normal biological processes of CD4^+^ T cells, and leads to the functional changes of CD4^+^ T cells, which is an underlying cause of systemic lupus erythematosus (SLE). In this review, we focus on the recent advances in the roles of ncRNAs in CD4^+^ T cell functions and differentiation, as well as their potential applications in the diagnosis and treatment of SLE.

## Introduction

Systemic lupus erythematosus (SLE) is a highly heterogeneous autoimmune disease triggered by a large number of autoantibodies and the formation of immune complexes, which cause multiple organ damage and serious disease outcomes. Therefore, in order to develop earlier diagnosis and more effective treatment, it is necessary to better understand the pathogenesis of SLE.

SLE is characterized by overactive T cells that differentiate into different T helper (Th) subsets secreting inflammatory cytokines and supporting B cells to produce autoantibodies. Naive CD4^+^ T cells in lupus undergo the proinflammatory shifts in epigenetics that favor T cell activation and effector T cell immune responses, which trigger lupus flares (1). Our previous studies demonstrate that several hypomethylated genes, such as CD70, CD40LG, ITGAL, and PRF1, are associated with the hyperactivity of T cells in SLE (2–4). The imbalance of Th cell subsets, particularly the deficient proportion of regulatory T cells and the abundance of effector T cells, including Th1, Th2, Th17, and Tfh cells, contributes to the development of SLE and correlates closely with the disease activity (5). The ratio of Th1/Th2 significantly increases in patients with SLE, which is related to the severity of renal damage (6). In addition, Th1 cells promote the secretion of IgG2a and IgG3 antibodies by secreting IFN-γ, thus accelerating the development of immune complex glomerulonephritis (7). Th17 cells exert its effector function mainly by secreting proinflammatory cytokine IL-17 that is increased in SLE patients, indicating the important role of Th17 cells in SLE (8). The phenotypic and functional heterogeneity of Treg cells is also involved in SLE development. Interestingly, the percentage of circulating CD4^+^ CD25^+^ Foxp3^+^ Treg cells decreases significantly in SLE and negatively correlates with the SLE Disease Activity Index (SLEDAI) score (9). CD4^+^ CD25^+^ Treg cells freshly isolated from SLE patients secrete increased proinflammatory cytokines IFN-γ and IL-10 and decreased anti-inflammatory cytokine TGF-β (9). On the contrary, circulating CD4^+^ CD25^−^ Foxp3^+^ Treg cells, with a pro-inflammatory feature and a deficiency in suppressive function, accumulate in SLE and correlate with the disease activity (10–12). In patients with SLE, peripheral CD4^+^ CXCR5^+^ PD-1^+^ Tfh cells (regarded as a GC-Tfh counterpart) are increased and are associated with a more serious disease phenotype, and CCR7^−^ PD-1^hi^ CXCR5^+^ CD4^+^ T cells (regarded as an activated Tfh cell phenotype) are associated with elevated autoantibody titers and the disease activity (13, 14). Imbalanced cytokine profiles in CD4^+^ T cell subsets, especially the predominance of inflammatory cytokines, are also involved in the pathogenesis of SLE (15). However, the mechanism that causes the aberrant activation, differentiation and function of CD4^+^ T cells in SLE remains largely unclear.

Non-coding RNAs (ncRNAs) are widely involved in the biological processes of immune cells by altering target gene expression. Mounting evidence has demonstrated that the abnormal expression of ncRNAs plays a key role in CD4^+^ T cell dysfunction and SLE development. Researches on the roles of ncRNAs in CD4^+^ T cell differentiation and functions provide new insights into the phenotypic and functional heterogeneity of CD4^+^ T cells, as well as the pathogenic mechanism of SLE. Furthermore, current studies focus on digging up available ncRNA biomarkers for the clinical management of SLE. Here, we review the current understanding of the roles of ncRNAs in CD4^+^ T cell differentiation and functions, as well as their potential applications in SLE.

## Classification and Functions of ncRNAs

Generally, the most widely studied ncRNAs are microRNAs (miRNAs), long non-coding RNAs (lncRNAs) and circular RNAs (circRNAs). Processed by the RNase III enzyme DROSHA and DICER, precursor miRNAs become mature miRNAs, a type of small (~21 nucleotides) endogenously expressed ncRNAs, which can post-transcriptionally suppress gene expression by binding to specific miRNA response elements (MREs) on their target transcripts (16). The canonical regulatory mechanism of miRNAs is binding to the site in 3′UTR of target mRNA through a form of RNA-induced silencing complex (RISC) containing specialized nuclear Argonaute (Ago) protein, resulting in mRNA degradation or translational inhibition (17). A single miRNA can tune the production of hundreds of proteins by directly or indirectly regulating thousands of genes, suggesting the profound and far-reaching implications of miRNAs on eukaryotic transcriptomes and proteomes (18). However, with a deeper understanding of the characteristics of miRNAs, researchers have uncovered the positive role of miRNAs in activating gene expression, thus making it more difficult to explain the regulatory mechanism of miRNAs. Previously, both Ago1 and Ago2 were considered to suppress gene expression (19). Nonetheless, a later research has reported that Ago2, but not Ago1, can also activate gene expression when its target mRNA lacks poly (A) tail, suggesting that the role of Ago2 is dependent on the length of mRNA poly(A) tail (20).

LncRNAs are a kind of ncRNAs more than 200 nucleotides and have various biological functions based on their structural characteristics and loci to the protein-coding genes (21). According to the location to protein-coding genes, lncRNAs can be classified into five categories, including long intergenic non-coding RNAs (lincRNAs), sense lncRNAs, anti-sense lncRNAs, bidirectional lncRNAs, intronic lncRNAs, each of them has potential in gene regulation. LncRNAs can regulate the expression of nearby protein-coding genes, also known as cis-regulatory action. For example, Wang et al. (22) found that chromosome looping brings lncRNA HOTAIR to the adjacent position of its target gene, and then HOTAIR targets WDR5/MLL complex by directly binding with WDR5, driving histone H3 lysine 4 trimethylation and gene transcription. Furthermore, lncRNAs are involved in adjusting the activity and localization of specific proteins, such as transcription factors. Specifically, lncRNA NRON can bind with the transcription factor NFAT and transcriptional regulators by targeting a scaffold protein to form an RNA-protein scaffold complex, which hinders the interaction between NFAT and phosphatases and prevents NFAT from dephosphorylation, thus inhibiting NFAT-mediated gene transcription (23). LncRNAs can act as miRNA sponges to regulate gene expression, which is also called competing endogenous RNA (ceRNA) mechanism (24, 25). For instance, linc-RoR acts as a miR-145 sponge to prevent Oct4, Sox2, and Nanog from miR-145-mediated degradation (26). In addition, when anti-sense lncRNA gene and protein-coding gene overlap, anti-sense lncRNA transcription inhibits the recruitment of RNA polymerase (Pol II) to the protein-coding gene promoter, leading to the transcriptional inhibition of the protein-coding gene (27).

CircRNAs, first identified in early 1991, are considered as a novel ncRNA subclass with a covalent-closed and stable loop structure (28). Pre-mRNAs undergo canonical splicing to form linear mRNAs, while circRNAs are produced when pre-mRNAs undergo backsplicing. Endogenous circRNAs are resistant to exonucleases-mediated degradation, which makes them get a long half-life and stably exist in mammals. Similar to lncRNAs, most circRNAs carry MREs and can serve as miRNA sponges to affect gene expression (25, 29). For instance, ciRS-7 acts as a miR-7 sponge to regulate Ago protein, and circular sex-determining region Y (Sry) acts as a miR-138 sponge to reserve miR-138-mediated inhibition of Ago2 (30). Besides, circRNAs can be miRNA “reservoirs” to stabilize the function of miRNAs. Hansen et al. (31) first reported this novel pattern of miRNA “reservoir” in the miR-7/miR-671/cisR-7 regulatory axis. In this signaling pathway, miR-671-mediated cleavage of cisR-7 results in the release of miR-7, resulting in the significant down-regulation of miR-7 targets. CircRNAs also control gene transcription through a special form of protein sponge. For example, circ-Foxo3 binds with functional proteins P21 and CDK1, forming a large circRNA-protein complex to regulate target gene transcription (32).

## The Role of ncRNAs in CD4^+^ T Cell Differentiation and Functions

### ncRNAs in Th1 Cells

Th1 cells are characterized as expressing the vital transcription factor T-bet and secreting effector cytokines IFN-γ and IL-10, which play a role in mediating chronic inflammatory responses against viruses, intracellular pathogens and tumors. Besides, STAT1 is crucial to IFN-γ signaling, and STAT4 is critical for IL-12 signaling, both of which are the key regulators for Th1 cell differentiation (33) (Table 1).

**Table 1:** The characteristics of CD4^+^ T subsets.

**CD4^**+**^ T subset**	**Activation by cytokine**	**Signaling**	**Master transcriptional factor**	**Produced cytokine**	**Function**
Th1	IL-12 and IL-10	SOCS1, STAT1, and STAT4	T-bet	IFN-γ, IL-10, and IL-12	Mediating chronic inflammatory response
Th2	N/A	NF-κB, STAT6, and PI(3)K	GATA-3	IL-4	Anti-helminth immunity, atopic asthma, IgE synthesis, and eosinophilia
Th17	IL-1β, IL6, IL-23, and TGF-β	SOCS1, SMAD7, and STAT3	RORγt	IL-17 and IL-21	Eliminating pathogens in host defense reactions and inducing tissue inflammation
Tfh	N/A	PI3K-mTOR, KLF2, PTEN, and ICOS-ICOSL	BCL6	IL-6 and IL-21	Supporting GC B cell differentiation into plasma cells and memory B cells
Treg	IL-2 and TGF-β	SOCS1, SMAD3, STAT3, STAT5, and mTOR	FOXP3	TGF-β	Maintaining immune homeostasis and self-tolerance

DICER-deficient T cells lose the ability to generate mature miRNAs and are inclined to differentiate into Th1 cells, suggesting the role of miRNAs in Th1 cell differentiation (34). Furthermore, several miRNAs, such as miR-21, and miR-29, are down-regulated in DICER-deficient CD4^+^ T cells (34). miR-29 limits the differentiation of Th1 cells and the production of IFN-γ by targeting T-bet and Eomes directly (35). Inhibiting miR-21 shifts the balance of Th1/Th2 toward Th1 cells by improving the secretion of IL-12 in dendritic cells (DCs) and NK cells (36). miR-148a controls Th1 cell survival by targeting the pro-apoptotic gene Bim, and the expression of miR-148a can be induced by Twist1 and T-bet, the critical transcription factors controlling Th1 cell fate (37, 38). Similarly, the overexpression of miR-142a-5p in activated lymphocytes contributes to T cell differentiation toward Th1 cells by targeting SOCS1 and TGFBR1 (39). miRNAs also play a part in regulating the migration and retention of Th1 cells. Deleting miR-31 promotes the expression of genes involved in T cell activation and chemotaxis, leading to the increased migratory ability of Th1 cells. Th1 transcription factor T-bet and FOXO1, respectively, act as positive and negative regulators for miR-31, indicating the interplay between miRNAs and cell signaling molecules (40). In addition, miRNAs can affect the propensity of cytokine production in Th1 cells. The differentiation of IL-10^+^ Th1 cells and IFN-γ^+^ Th1 cells are reciprocally restricted, as the increased IL-10 secreted by Th1 cells limits the differentiation of IFN-γ-secreting Th1 cells (41). miR-150 promotes IL-10-secreting Th1 cell differentiation by targeting SLC2A1 and modulating glucose uptake. However, the expression of miR-150 is decreased in IFN-γ-secreting Th1 cells, suggesting that miR-150 serves as a switch to promote IL-10^+^ Th1 cell differentiation and inhibit IFN-γ secretion (42). LncRNA-Ifng-AS1, also named NeST or Tmevpg1, is essential for the development of Th1 cells. Collier et al. (43) found that Ifng-AS1 and its human ortholog IFNG-AS1 are located near the IFN-γ encoding gene Ifng. LncRNA-Ifng-AS1 cooperates with T-bet or other critical factors to promote Ifng expression, but lncRNA-Ifng-AS1 alone is insufficient for regulating Ifng gene transcription. The abnormal expression of IFNG-AS1 in Th1 cells also correlates with several autoimmune disorders, such as multiple sclerosis (MS) and Hashimoto's Thyroiditis (HT) (44, 45) (Table 2).

**Table 2:** ncRNAs involved in Th1 cells.

**ncRNA**	**Target gene**	**Biological function**	**Reference**
miR-21	N/A	Inhibits the differentiation of Th1 cells by regulating IL-12 secretion	(36)
miR-29	T-bet and Eomes	Promotes the differentiation of Th1 cells	(35)
miR-148	Bim	Contributes to Th1 cell development	(37, 38)
miR-142a-5p	SOCS1 and TGFBR1	Promotes the differentiation of Th1 cells	(39)
miR-31	T-bet and FOXO1	Negatively regulates T cell activation and migratory activity of Th1 cells	(40)
miR-150	SLC2A1	Promotes IL-10^+^ Th1 cell differentiation	(42)
LncRNA-Ifng-AS1 (NeST, Tmevpg1)	Ifng	Promotes the differentiation of Th1 cells	(43)

### ncRNAs in Th2 Cells

Th2 cells secrete the master functional cytokine IL-4 and play a critical role in mediating IgE synthesis, eosinophilia, anti-helminth immunity, and atopic asthma. GATA-3, the central regulator of Th2 cells, is necessary and sufficient for the expression of IL-4 in CD4^+^ T cells, which further activates STAT6 to inhibit Th1 cell differentiation, thus determining the commitment to Th2 phenotype (46) (Table 1).

The miRNA expression profiling of human airway-infiltrating CD4^+^T cells reveals that miR-19, a member of the miR-17~92 clusters, is highly expressed in asthma, and cells lacking miR-17~92 clusters are compromised in terms of Th2 cell-mediated responses. Functionally, miR-19 facilitates Th2 cell-related cytokine production by targeting PTEN, SOCS1 and A20 to amplify NF-κB, JAK-STAT and PI(3)K signaling pathways (47). miR-23~27~24 clusters also play an important part in Th2-mediated immune responses. miR-24 and miR-27 collaboratively inhibit the differentiation of Th2 cells and the production of functional cytokine IL-4. miR-27 limits IL-4 production by directly repressing the transcription factor GATA-3. However, other direct targets of miR-24 and miR-27, including Cnot6, Clcn3, Ikzf1, Gpr174, and Galnt3, have few effects on IL-4, but they may alter the expression of molecules related to bypassing signaling pathways (48, 49). In addition, Th2 cells lacking miR-155 exhibit a significant deficiency in migrating ability. In-depth study finds that miR-155 can target S1pr1 to promote Th2-mediated airway allergy (50) (Table 3).

**Table 3:** ncRNAs involved in Th2 cells.

**ncRNA**	**Target gene**	**Biological function**	**Reference**
miR-19	Pten, Socs1, and A20	Augments the cytokine production of Th2 cells	(47)
miR-23~27~24	GATA-3	Inhibits the production of IL-4 and the differentiation of Th2 cells	(48, 49)
miR-155	S1pr1	Contributes to the migration of Th2 cells	(50)

### ncRNAs in Th17 Cells

Th17 cells are characterized by producing functional cytokines IL-17 and IL-21, as well as expressing the central transcription factor RORγt. TGF-β serves as a key regulator for the differentiation and function of Th17 cells by inducing RORγt expression (51). Th17 cell-mediated host defense plays a critical role in eliminating pathogens, however, the uncontrolled immune responses can also be a trigger for tissue inflammations and autoimmune diseases (52) (Table 1).

Mounting evidence has demonstrated that miRNAs are involved in Th17 cell-mediated inflammatory responses and autoimmunity. For instance, miR-155 facilitates the differentiation and function of Th17 cells by targeting SOCS1 (53). Moreover, miR-155 drives DCs to produce pro-inflammatory cytokines crucial for the differentiation of Th17 cells (54). Mice conditionally knocking out miR-223 in myeloid dendritic cells (mDCs) exhibit increased expression of PD-L1 and decreased levels of pro-inflammatory cytokines IL-1β, IL-6, and IL-23 necessary for driving pathogenic Th17 cell differentiation, thus slowing the progression of experimental autoimmune encephalomyelitis (EAE) (55). Similarly, mice lacking miR-21 are deficient in Th17 cell differentiation and are resistant to EAE. Functionally, miR-21 suppresses the expression of smad7, a negative regulator of TGF-β, which further activates the downstream SMAD-2/3 signaling pathway that induces TGF-β expression, thus contributing to Th17 cell differentiation (56). Dicer1-regulated microRNA-183-96-182 clusters (miR-183C) contribute to the pathogenicity of Th17 cells in autoimmunity. Deleting Dicer1, an encoding gene responsible for the biogenesis and processing of miRNAs, inhibits the production of inflammatory cytokines in Th17 cells, suggesting the critical role of miRNAs in regulating the pathogenic function of Th17 cells. Furthermore, the expression of miR-183C significantly increases in Th17 cells under the induction of IL-6-STAT3 signaling. Enhanced miR-183C further promotes the production of pathogenic cytokines in Th17 cells via suppressing Foxo1, a negative transcriptional factor of Th17 cells, resulting in serious autoimmune diseases (57). The increased expression of miR-210 induced by hypoxia plays a key role in Th17 cell differentiation. Under normoxic conditions, hypoxia-inducible factor 1α (HIF-1α) is proline hydroxylated by prolyl hydroxylases (PHDs), and then hydroxylated HIF-1α is recognized by the von Hippel Lindau protein (VHL) E3 ligase complex, resulting in HIF-1α ubiquitination and proteasomal degradation (58). On the contrary, hypoxic conditions protect HIF-1α from VHL-mediated ubiquitination, thus promoting its expression. HIF-1α expresses shortly after T cell activation, which initiates metabolic reprogramming and the transcription of Th17 lineage-defining transcription factor RORγt. The expression of miR-210 induced by HIF-1α occurs late in Th17 differentiation, which limits the function of HIF-1α via negative feedback. Under hypoxic conditions, inhibiting miR-210 promotes the differentiation of Th17 cells by enhancing the expression of HIF-1α (59). LincRNA-p21, a kind of hypoxia-induced lncRNAs, inhibits VHL-mediated HIF-1α ubiquitination by binding with HIF-1α and VHL and disturbing their interaction, thus contributing to HIF-1α accumulation. Besides, lincRNA-p21 and HIF-1α can form positive feedback on HIF-1α expression and HIF-1α-dependent glycolysis (60). Furthermore, lncRNA 1700040D17Rik is significantly decreased in EAE mouse model, while highly expressed 1700040D17Rik is observed in mice after treated by rhIL23R-CHR. Functional study demonstrates that 1700040D17Rik is involved in Th17 cell differentiation by regulating RORγt expression (61) (Table 4).

**Table 4:** ncRNAs involved in Th17 cells.

**ncRNA**	**Target gene**	**Biological function**	**Reference**
miR-155	SOCS1	Enhances the differentiation of Th17 cells and the production of IL-17	(53)
miR-223	Roquin-1	Regulates mDCs-induced Th17 cell differentiation	(55)
miR-21	Smad7	Promotes the differentiation of Th17 cells through smad/TGF-β signaling pathway	(56)
miR-183C	Foxo1	Promotes the differentiation of Th17 cells	(57)
miR-210	HIF-1α	Inhibits Th17 cell differentiation	(59)
LincRNA-p21	HIF-1α	Stabilizes HIF-1α expression and function	(60)
LncRNA- 1700040D17Rik	RORγt	Inhibits the differentiation of Th17 cells	(61)

### ncRNAs in Follicular Helper T Cell (Tfh) Cells

Tfh cell differentiation depends on the expression of lineage-defining transcription factor BCL6. Several characteristic molecules, such as CXCR5, PD-1, BTLA4, ICOS, IL-21, and SAP, also play important roles in the differentiation and function of Tfh cells, and they are indispensable for supporting the development of GC B cells (62). Generally, Tfh cell differentiation can be divided into two consecutive stages. The first stage is pre-GC Tfh cells, which is initialed by antigen-presenting cells (APCs). Under the stimulation from APCs, pre-GC Tfh cells migrate to the border of germinal centers (GCs), where the interaction of T/B cells occurs, and then highly express surface markers CXCR5, PD-1, ICOS, and SAP, which are necessary to determine the molecular basis for commitment to Tfh cell phenotype and promote GC formation (63, 64). The second stage is GC Tfh cells, which mainly participate in regulating GC responses and promote the differentiation of GC B cells into plasma cells and memory B cells. In GC Tfh cells, several important molecules, including CXCR5, PD-1, BCL6, BTLA4, ICOS, and SAP, reach a peak, while CD127, PSGL1, and EBI2 decline (64–66) (Table 1).

miRNAs are important for post-transcriptionally regulating Tfh cell differentiation in different stages. miR-17~92 plays a key role in the early stage differentiation of Tfh cells (67, 68). miR-17~92 facilitates robust Tfh cell differentiation after initial T cell activation by repressing Rora and other inappropriate genes (69). Interestingly, miR-17~92 induces the differentiation of CXCR5^+^ PD1^hi^ Tfh cells without increasing BCL6 expression, which is consistent with the phenotype of pre-GC Tfh cells. Furthermore, miR-17~92 controls the migration of Tfh cells to B follicles by enhancing ICOS-PI(3)K signaling via repressing Akt phosphatase Phlpp2, an inhibitor for PI3K-Akt-mTOR signaling pathway (68). The RNA binding protein Roquin inhibits spontaneous T cell activation and Tfh cell differentiation via post-transcriptional regulation and plays a part in maintaining peripheral tolerance and preventing autoimmune diseases (70). Functionally, Roquin inhibits the PI3K-mTOR signaling pathway by preventing miR-17~92 binding with 3'UTR of PTEN, resulting in the upregulation of PTEN and the decreased differentiation of Tfh and Th17 cells (71). However, miR-92a, a member of miR-17~92 clusters, promotes Tfh cell development by inhibiting KLF2 and PTEN in human autoimmunity, indicating the variability of miRNA functions in different immune contexts (72). The transcription factor BCL6 can also affect miRNA expression to exert its regulatory role on Tfh cells. miR-31 limits the function of human Tfh cells by inhibiting the expression of molecules relevant to Tfh cell biology, such as SAP and CD40L. In human Tfh cells, miR-31 is specifically and strongly down-regulated by BCL6 (73). miR-155 is rapidly induced in activated T cells, which overlaps with the expression kinetics of miR-17~92 in activated CD4^+^ T cells. Consistently, deleting miR-155 in T cells compromises the early Tfh cell differentiation and expansion (74). Moreover, miR-155 promotes the proliferation of Tfh cells especially at the late stage of Tfh cell differentiation and positively controls CD40L expression in antigen-specific CD4^+^ T cells by suppressing the Peli1-c-Rel signaling pathway (75). Furthermore, the miR-155–Peli1–c-Rel pathway is important to Tfh cell differentiation but is dispensable for non-Tfh cell differentiation, highlighting its specific role for Tfh cell lineage (75). On the contrary, miR-146a plays a negative part in Tfh cell generation. miR-146a is induced by TCR-driven NF-κB activation and serves as a negative regulator in antigen-specific T cell response *in vivo*, for both primary and recall responses (76). The expression of miR-146a highly increases in Tfh and GC B cells, and peaks in the late stage of GC responses, coinciding with the decline of Tfh cell-mediated immune responses after immunization. miR-146a can repress multiple Tfh-related mRNAs, especially genes related to the ICOS-ICOSL signaling pathway in Tfh and GC B cells, thus inhibiting Tfh and GC B cell accumulation, which is important to maintain immunological tolerance and prevent autoimmune diseases (77). Besides, miR-146a-deficient T cells are hyperactive in chronic inflammation and are involved in T cell-mediated autoimmunity (76). Similarly, miR-146b, a paralog of miR-146a, also acts as an inhibitor of Tfh and Th17 cell differentiation (78). In addition, lncRNA Malat1 is highly expressed in T cell subsets and is widely involved in immune responses by regulating immune gene expression. Although the deficiency of Malat1 in T cells does not influence the differentiation and B cell helper function of Tfh cells, inhibiting Malat1 in monocytes can reduce the expression of proinflammatory cytokine IL-21 (79) (Table 5).

**Table 5:** ncRNAs involved in Tfh cells.

**ncRNA**	**Target gene**	**Biological function**	**Reference**
miR-17~92	Rora and PHLPP2	Promotes the differentiation and migration of Tfh cells	(69, 71)
miR-92a	KLF2 and PTEN	Promotes Tfh cell development	(72)
miR-31	CD40L and SAP	Inhibits the ability of Tfh cells to support B cells	(73)
miR-155	Peli1	Promotes the differentiation of Tfh cells and the expression of CD40L	(75)
miR-146a and miR-146b	ICOS and CD40	Regulate ICOS–ICOSL and CD40-CD40L signaling to limit the accumulation of Tfh cells and GC responses	(77, 78)
Lnc-Malat1	N/A	Increases in T cells and plays a positive role in secreting proinflammatory cytokine IL-21	(79)

### ncRNAs in Treg Cells

Treg cells characteristically express CD25 and the lineage-specific transcription factor Foxp3. CD25 is an original marker of Treg cells, which represents an activated state of CD4^+^ T cells, and Foxp3 determines the differentiation and suppressor function of Treg cells. Foxp3 expression is initialed by IL-2 signaling and influenced by particular TCR signaling and TGF-β (80). Furthermore, IL-2 receptor beta-dependent STAT5 activation is necessary for Foxp3 expression and Treg cell development (81). TGF-β is also responsible for balancing the reciprocal conversion between Th17 and Treg cells (82, 83). Foxp3^+^ Treg cells suppress overactive immune responses to maintain immune homeostasis and inhibit autoreactive T cells to preserve self-tolerance, whereas the dysfunction of Treg cells can cause autoimmune disorders and tumor immune escape (Table 1).

ncRNAs play a critical role in controlling the differentiation and function of Treg cells. The deficiency of miR-31 in CD4^+^ T cells promotes Treg cell development, leading to the alleviation of EAE and Ang II (Angiotensin II)-induced hypertension in mice, while the overexpression of miR-31 inhibits Treg cell differentiation by targeting Foxp3, Gprc5a, and Protein phosphatase 6c (Ppp6C) that are indispensable for Treg cell development (84, 85). On the contrary, miR-125a stabilizes the immunoregulatory capacity of Treg cells by suppressing several effector T lineage genes, including Stat3, Ifng, and Il13. miR-125a-deficient mice are inclined to develop more severe symptoms of EAE, which are alleviated after rescuing miR-125a (86). In the setting of a limited number of IL-2, miR-155 is required for maintaining the competitive fitness of Treg cells. Foxp3 controls the function of Treg cells by driving miR-155 expression, thus contributing to the reduced expression of SOCS1 protein and the increased level of high-affinity IL-2R in Treg cells (87). Treg cells contain a high level of cyclic AMP (cAMP) necessary for maintaining its suppressive function. However, upregulated miR-142-3p can restrict the level of cAMP in CD4^+^T cells, thus compromising the suppressive function of Treg cells (88). Interestingly, miR-142-5p, another mature isoform of miR-142, plays a positive role in regulating cAMP-mediated suppressor function of Treg cells. Deleting miR-142-5p decreases the concentration of intracellular cAMP by targeting AMP-hydrolyzing enzyme phosphodiesterase-3b (Pde3b), leading to the deficient function of Treg cells and immune disorder in mice despite preserving a number of Treg cells with a normal phenotype (89). miR-99a and miR-150 can target mTOR, which is responsible for cell growth, to promote the differentiation of Treg cells at the expense of inhibiting Th17 cells (90). miR-99a and miR-150 suppress the expression of mTOR by binding two adjacent target sites in it. Besides, an intact target site of miR-99a is necessary for miR-150-mediated regulation to mTOR, because miR-150 activation depends on miR-99a binding to its adjacent target site of mTOR (90).

Recently, scientists have uncovered a distinctive lncRNA transcriptome of Treg cells. And lncRNA Flatr, a core member of the lncRNA transcriptome of Treg cells, promotes Treg cell development by enhancing the expression of Foxp3 (91). LncRNA-smad3 also plays a role in controlling Treg cell fate and regulating T cell-mediated autoimmunity. Functionally, lnc-smad3 recruits histone deacetylase HDAC1 to the smad3 promoter, which silences smad3 transcription by altering smad3 locus accessibility, leading to the reduced expression of Foxp3 in Treg cells. Conversely, TGF-β activation inhibits lnc-smad3 expression in Treg cells, therefore promoting Foxp3 expression via restoring the accessibility of smad3 (92). LncRNA Flicr adjacent to Foxp3 in mouse and human genomes can affect the chromatin accessibility of Foxp3. Flicr-deficient mice show resistance to autoimmune diabetes, indicating that Flicr-deficient Treg cells may more effectively function as an immune suppressor than normal Treg cells (93) (Table 6).

**Table 6:** ncRNAs involved in Treg cells.

**ncRNA**	**Target gene**	**Biological function**	**Reference**
miR-155	SOSC1	Promotes the expression of IL-2R by down-regulating SOSC1	(87)
miR-142-3p	AC9	Limits the function of Treg cells by reducing the levelof intracellular cAMP	(88)
miR-142-5p	Pde3b	Decreases the concentration of intracellular cAMP and leads to Treg cell dysfunction	(89)
miR-31	Foxp3, Gprc5a, and Ppp6C	Inhibits Treg cell differentiation	(84, 85)
miR-125a	Stat3, Ifng, and Il13	Facilitates the differentiation and immune regulatory role of Treg cells	(86)
miR-99a and miR-150	Mtor	Promotes Treg cell differentiation at the expense of inhibiting Th17 cells	(90)
Lnc-Flatr	Foxp3	Promotes Treg cell development by enhancing Foxp3	(91)
Lnc-smad3	Smad3	Inhibits the expression of Foxp3 by suppressing smad3	(92)
Lnc-Flicr	Foxp3	Influences the chromatin accessibility of Foxp3	(93)

## The Roles of ncRNAs in the Aberrant Activation and Differentiation of CD4^+^ T Cells in SLE

The abnormal activation and differentiation of CD4^+^ T cells cause the pathogenic expansion of Th1/Th17/Tfh cells and Treg cell deficiency, which are implicated in SLE development. However, the regulatory mechanism leading to CD4^+^T cell dysfunction in SLE remains largely unknown. ncRNAs play an important role in CD4^+^ T cell activation and differentiation. Many studies have identified that the expression of above-mentioned ncRNAs, such as miR-21, miR-155, miR-31, miR-29a, miR-126, miR-142-3p/5p, and miR-183C, as well as several newly discovered lncRNAs and circRNAs, are markedly altered in the CD4^+^ T cells or peripheral blood mononuclear cells (PBMCs) of SLE, and are involved in SLE development by directly or indirectly regulating the aberrant activation and differentiation of CD4^+^ T cells.

### ncRNAs Regulate the Over-Activation of CD4^+^ T Cells in SLE

ncRNAs are implicated in regulating lupus CD4^+^ T cell over-activation. miR-148a and miR-21 can target DNA methyltransferase 1 (DNMT1) to promote the DNA demethylation of CD70 and LFA-1 that are responsible for T cell hyperactivity (94). Likewise, miR-29a facilitates the expression of immune gene CD70 and CD11a in lupus CD4^+^ T cells by targeting sp1 to repress DNMT1 (95). Our previous studies identify several miRNAs, including miR-126 and miR-142-3p/5p that are involved in CD4^+^ T cell over-activation in SLE (96–98). We found that upregulated miR-126 promotes the expression of CD11a and CD70 in lupus CD4^+^ T cells via inhibiting DNMT1-mediated DNA methylation (96). In addition, we also reported that the decreased expression of miR-142-3p/5p in lupus CD4^+^ T cells is involved in T cell hyperactivity and B cell hyperstimulation by targeting SAP, CD84, and IL-10 (97). Enhancer of zeste homolog 2 (EZH2), an epigenetic modulator important to T cell activation and differentiation, is over-activated in lupus CD4^+^ T cells. Coit et al. (1) found that the reduced expression of miR-26a and miR-101 is responsible for the increased activity of EZH2 in lupus CD4^+^T cells, which causes the epigenetic reprogramming that favors T cell activation and effector T cell differentiation, leading to lupus flares.

Recently, Chen et al. have reported that circRNAs form small imperfect RNA duplexes to bind the protein kinase (PKR) and inhibit its activation, while circRNAs are mostly degraded by a PKR-dependent manner. Improving the level of dsRNA-containing circRNAs suppresses the aberrant activation of PKR in lupus T cells, indicating the role of circRNAs in controlling PKR activity in SLE (99). It was reported that RANTES is involved in the development of SLE, and the transcriptional factor KLF13 regulates the expression of RANTES in activated T cells (100). Zhang et al. (101) demonstrated that hsa_circ_0012919 is highly expressed in lupus CD4^+^ T cells and participates in regulating the expression of RANTES and KLF13 by serving as the miR-125a-3p sponge. Moreover, inhibiting hsa_circ_0012919 corrects the DNA hypomethylation of CD70 and CD11a in lupus CD4^+^T cells by promoting DNMT1 activity (101). The AKT signaling is over-activated in CD4^+^ T cells from SLE patients (102–104). And miR-29a regulates the AKT signaling pathway in lupus CD4^+^ T cells by indirectly targeting DNMT1 (95). Wang et al. (105) found that circIBTK is down-regulated in SLE and negatively correlates with the SLEDAI score and anti-double-stranded (ds)DNA antibodies but positively correlates with the level of complement component 3 (C3) in patients with SLE. CircIBTK serves as the miR-29b sponge, which may inhibit the activation of the AKT signaling pathway in lupus CD4^+^ T cells by sponging miR-29a. LncRNAs also play a role in CD4^+^ T cell over-activation in SLE. LncRNA UCA1 is highly expressed in the PBMCs of SLE patients and positively correlates with the SLEDAI score and the levels of C3 and anti-dsDNA antibodies. Furthermore, UCA1 promotes the expression of AKT and PI3K correlated with SLE progression (106) (Table 7).

**Table 7:** The roles of ncRNAs in the aberrant activation of CD4^+^ T cells in SLE.

**ncRNA**	**Function**	**Mechanism**	**Reference**
miR-148a and miR-21	T cell hyperactivity	Targets DNMT1 to regulate CD70 and LFA-1	(94)
miR-29a	T cell hyperactivity	Targets Sp1 to regulate DNMT1, CD70, and CD11a	(95)
miR-126	T cell hyperactivity	Targets DNMT1 to regulate CD70 and CD11a	(96)
miR-142-3p/5p	T cell hyperactivity	Targets SAP, CD84, and IL-10	(97)
miR-26a and miR-101	T cell hyperactivity and non-Th1 effector T cell differentiation	Target EZH2 to regulate Th2-, Th17-, and Tfh-related genes	(1)
dsRNA-containing circRNAs	T cell activation	Inhibits PKR signaling	(99)
hsa_circ_0012919	T cell activation	Promotes the DNA demethylation of CD70 and CD11a and serves as the miR-125-3p sponge to regulate KLF13 and RANTES	(101)
circIBTK	T cell activation	Serves as the miR-29a sponge to regulate AKT signaling	(105)
LncRNA UCA1	T cell activation	Promotes AKT activation	(106)

### ncRNAs Regulate the Aberrant Differentiation of CD4^+^ T Subsets in SLE

MiR-155 is dysregulated in CD4^+^ T cells from both patients with SLE and lupus-prone mice. Divekar et al. (107) found that in lupus-prone mice, the expression of miR-155 significantly increases in Treg cells with an abnormal phenotype (CD62L^−^CD69^+^) and a deficient suppressive capability. Functionally, miR-155 targets CD62L to alter the phenotype of Treg cells in lupus-prone mice, suggesting that miR-155 is involved in the phenotypic heterogeneity of Treg cells. Deleting miR-155 limits the proportion of activated T cells and the levels of inflammatory cytokines IFN-γ, IL-4 and IL-17 secreted by Th1, Th2, and Th17 cells, thus alleviating the disease severity of lupus-prone mice (108). Diffuse alveolar hemorrhage (DAH) is a severe complication of SLE. Zhou et al. (109) reported that down-regulating miR-155 corrects the abnormal activation of NF-κB signaling and ameliorates the lung inflammation of pristane-induced DAH. On the contrary, Lashine et al. (110) found that the expression of miR-155 decreases in PBMCs from juvenile SLE and negatively correlates with the disease activity and proteinuria but positively correlates with the white blood cell (WBC) count. Besides, correcting the expression of miR-155 represses the expression of phosphatase 2A (PP2A) and promotes the secretion of IL-2 in PBMCs from juvenile SLE patients. To sum up, as SLE is a highly heterogeneous autoimmune disease, the differences in patients and disease stages probably count for the contradiction in the expression and role of miR-155 in SLE.

miR-21 is upregulated in both PBMCs or CD4^+^T cells from SLE patients and splenic CD4^+^ T cells from lupus-prone mice (111, 112). miR-21 contributes to aberrant CD4^+^ T cell responses in human SLE by targeting PDCD4, while inhibiting miR-21 *in vitro* improves abnormal CD4^+^ T cell responses, including the production of IL-10, and the expression of CD40L, as well as the capability to support B cell development, which indicates the pathogenic role of miR-21 in SLE (113). Garchow et al. (114) reported that miR-21 deficiency protects mice from chronic graft-versus-host disease (cGVHD)-induced lupus by regulating Th17/Treg balance. They observed that cGVHD-affected mice with miR-21 deficiency have a reduced proportion of Th17 cells and an expanded compartment of Treg cells. Moreover, miR-21 inhibits the CD40: CD40L and CD28: CD80/86 co-stimulation pathways that are involved in establishing T cell tolerance and limiting adaptive immune response (114). Silencing miR-21 with a tiny seed-targeting LNA alleviates splenomegaly in B6.Sle123 mice and decreases the expression of PDCD4 and the population of Fas receptor-expressing lymphocytes (115). In SLE patients, the expression of miR-21 positively correlates with the disease activity and negatively correlates with the levels of C3 and IL-2, while the expression of miR-31 decreases and positively correlates with IL-2 production (116, 117). And IL-2 deficiency is responsible for the compromised suppressor ability of Treg cells in SLE (118). Furthermore, miR-21 and miR-155 are highly expressed in PBMCs from SLE patients with active nephritis and are the risk factors for lupus nephritis (LN), suggesting that miR-21 and miR-155 are potential biomarkers for assessing the severity of LN (119).

In addition to miR-155, miR-21, and miR-31, other miRNAs are also reported to be involved in aberrant differentiation of CD4^+^ T cell subsets in SLE, including miR-183C, miR-17-92 and miR-873. miR-183C is upregulated in both PBMCs from SLE patients and splenocytes from MRL/lpr mice (112). Interestingly, Li et al. (120) found that miR-183C is decreased in LN patients. Although it was reported that the increased expression of miR-183C plays a positive role in driving pathogenic Th17 cell differentiation and autoimmune diseases (57), their studies showed that overexpressing miR-183C in lupus mice reverses the ratio of Th17/Treg by regulating mTOR signaling, thus improving the severity of LN and the survival rate of lupus mice (120). miR-17-92 is critical for Tfh cell differentiation and is implicated in autoimmune diseases (68, 121). In addition, the members of miR-17-92 clusters (except miR-92a) are upregulated in CD4^+^ T cells from MRL/lpr mice and patients with SLE (122, 123). Upregulating miR-17-92 in mice facilitates the spontaneous activation and abnormal expansion of CD4^+^ T cells, and skews CD4^+^T cell differentiation toward Tfh cells, thus contributing to lymphoproliferative diseases and SLE-like symptoms (68, 124). In patients with SLE, the expression of miR-17, miR-19b and miR-20a is positively correlated with the SLEDAI score, and miR-17 and miR-20a are also positively associated with the titers of anti-dsDNA antibodies. Besides, miR-17 and miR-20a are negatively correlated with the level of C3, suggesting the different roles of miR-17-92 cluster members in indicating the disease activity of SLE (123). miR-873 is upregulated in SLE patients and positively correlates with the disease severity of SLE, and CD4^+^ T cells are the major source of miR-873 expression. Furthermore, miR-873 contributes to Th17 cell differentiation by targeting forkhead box O1 (Foxo1). Inhibiting miR-873 *in vivo* decreases the secretion of IL-17A and alleviates the progression of spontaneous SLE in MRL/lpr mice (125). Shao et al. reported that miR-34a limits the differentiation of Treg cells by inhibiting Foxp3 in CD4^+^ T cells. They found that the level of murine miR-34a decreases in TGF-β induced Treg cells but increases in Th17 cells induced *in vitro*. Moreover, the level of miR-34a positively correlates with the Th17 lineage-specific transcription factor RORγt but negatively correlates with the mRNA expression of FOXP3 in PBMCs of SLE, which indicates that miR-34a may play a part in the imbalance of Th17/Treg in SLE (126). In Treg cells of new-onset SLE, the expression of miR-326 is increased and negatively correlates with the mRNA expression of Ets-1 and positively correlates with the levels of CRP and anti-C1q antibodies from new-onset SLE patients, suggesting that miR-326 may regulate Treg cell dysfunction via targeting Est-1 in SLE (127).

**Figure 1:**
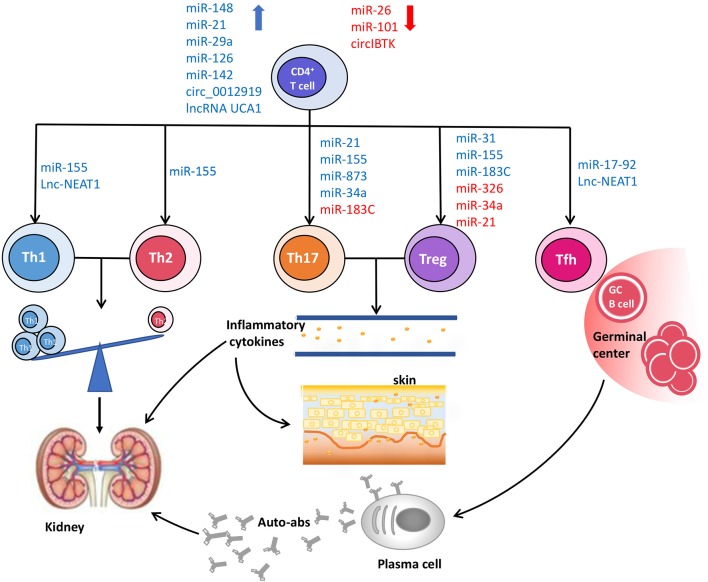
The roles of ncRNAs in CD4^+^ T cell dysfunction and pathogenesis of SLE. The aberrant expression of ncRNAs disturbs the normal biological processes of CD4^+^ T cells, and then leads to the phenotypic and functional changes of CD4^+^ T cells, including the imbalance of Th1/Th2, increased proportions of pro-inflammatory Th17 cells and Tfh cells, as well as deficient frequency and function of Treg cells, which contribute to the occurrence and development of SLE.

LncRNA-NEAT1 is highly expressed in PBMCs from SLE patients and positively correlates with the SLEDAI score (128). Inhibiting NEAT1 in LN patients reduces the expression of a group of chemokines and cytokines, including IL-6, CXCL10, CCL8, etc., which are involved in glomerular Th1 cell predominance (129). IL-6 is also important to support CD4^+^ T cell expansion and Tfh cell differentiation and functions as a major proinflammatory cytokine in SLE (15, 130). Furthermore, NEAT1 regulates a set of genes, such as IFI27, OAS1, CXCL9, IFI35, CXCL10, and CXCL11, involved in abnormal type I interferon signaling pathway in SLE (128) (Table 8) (Figure 1).

**Table 8:** The roles of ncRNAs in the aberrant differentiation of CD4^+^ T cells in SLE.

**ncRNA**	**Regulated CD4^**+**^ T cell subset**	**Molecular mechanism**	**Reference**
miR-155	Treg cells	Targets CD62L to alter the phenotype of Treg cells	(107)
miR-155	Th1, Th2, and Th17 cells	Targets S1PR1 to regulate the production of IFN-γ, IL-4, and IL-17A	(108)
miR-21	Th17/Treg balance	Inhibits CD40:CD40L and CD28:CD80/86 signaling pathways to destroy T cell tolerance	(114)
miR-31	Treg cells	Plays a role in IL-2 deficiency	(116)
miR-183C	Th17/Treg balance	Targets mTOR signaling to regulate Th17/Treg cell balance	(120)
miR-17-92	Tfh cells	Regulates transcriptional factor BCL6 or Pten and Bim	(68, 124)
miR-873	Th17 cell	Targets Foxp1 to promote the differentiation of Th17 cells and the production of IL-17A	(125)
miR-34a	Th17/Treg balance	Inhibits Treg cell differentiation by suppressing Foxp3 and promotes Th17 cell differentiation by down-regulating RORγt	(126)
miR-326	Treg cells	Targets Est1 to regulate Treg cell differentiation	(127)
LncRNA-NEAT1	Th1 and Tfh cells	Regulates Th1 cell related-chemotactic factor receptor CXCL10, CCL8, and Tfh cell-related cytokine IL-6	(128)

## Conclusion and Outlook

In summary, the functions and roles of many miRNAs are demonstrated in regulating CD4^+^T cell activation and differentiation and the processes of SLE. However, the specific functions of lncRNAs and circRNAs in CD4^+^ T cell development and their roles in the pathogenesis of SLE are largely unknown. The functional studies about circRNAs and lncRNAs mainly arrive from cancer disease and anti-tumor immunity. Using RNA-seq, researchers have discovered distinctive expression profiles of circRNAs in different immune cells of SLE, but the functions and mechanisms of those circRNAs in SLE are unclear. The future of ncRNA researches in the immune system is both promising and challenging, since the reported ncRNAs are just the tip of the iceberg in the human genome, and most of their functions need to be investigated by experimental methods. Studying the roles of ncRNAs in CD4^+^ T cell differentiation and their applications in SLE will help us to understand how ncRNA disorders cause CD4^+^ T cell dysfunction and the pathogenesis of SLE, thus paving the way for the development of diagnostics and therapeutics of SLE.

## Author Contributions

XG wrote the manuscript. LL, XM, and SJ edited the manuscript. MZ revised the manuscript.

### Conflict of Interest

The authors declare that the research was conducted in the absence of any commercial or financial relationships that could be construed as a potential conflict of interest.
